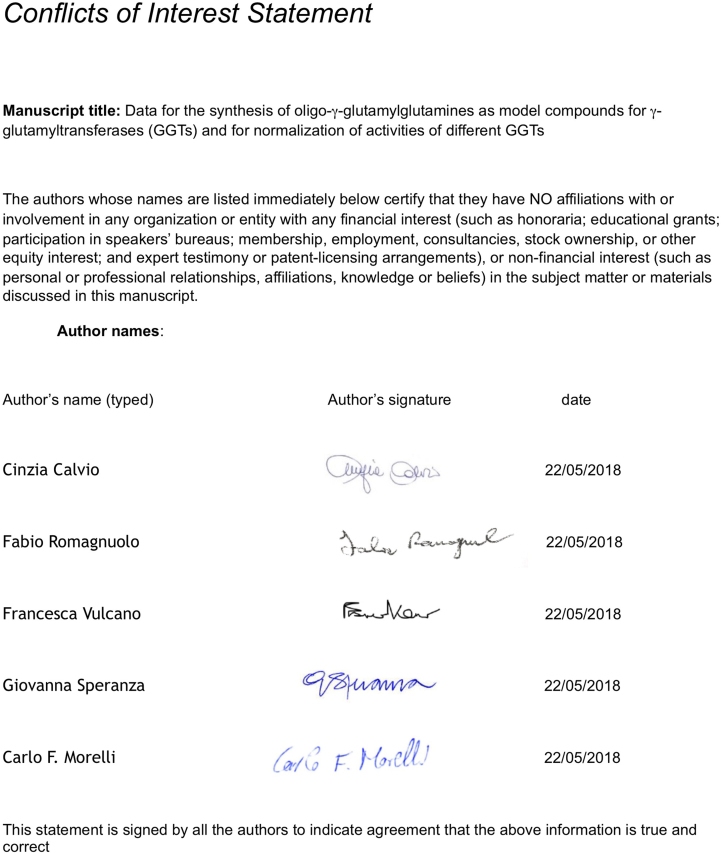# Data for the synthesis of oligo-γ-glutamylglutamines as model compounds for γ-glutamyltransferases (GGTs) and for normalization of activities of different GGTs

**DOI:** 10.1016/j.dib.2018.09.116

**Published:** 2018-10-06

**Authors:** Cinzia Calvio, Fabio Romagnuolo, Francesca Vulcano, Giovanna Speranza, Carlo F. Morelli

**Affiliations:** a Department of Biology and Biotechnology, University of Pavia, via Ferrata, 9, 27100 Pavia, Italy; b Italian Interuniversity Consortium on Materials, Science and Technology (INSTM), Pavia research Unit, 27100 Pavia, Italy; c Department of Chemistry, University of Milano, via Golgi 19, 20133 Milano, Italy; d Institute of Molecular Science and Technologies (ISTM-CNR), via Golgi 19, 20133 Milano, Italy

## Abstract

γ-Glutamyltransferases (GGTs) are widespread, conserved enzymes that catalyze the transfer of the γ-glutamyl moiety from a donor substrate to water (hydrolysis) or to an acceptor amino acid (transpeptidation) through the formation of a γ-glutamyl enzyme intermediate.

Although the vast majority of the known GGTs has a short sequence called lid-loop covering the glutamate binding site, *Bacillus subtilis* GGT and some other enzymes from *Bacillus* spp. lack the lid loop. In order to assess the possible role of the lid loop of GGTs in substrate selection, synthetic oligo-γ-glutamylglutamines containing up to three γ-glutamyl residues were used as model substrates. The activities of the enzymes under investigation were standardized with respect to a common reaction to ensure comparable results. The activity of an engineered mutant enzyme containing the amino acid sequence of the lid loop from *Escherichia coli* GGT inserted into the backbone of *B. subtilis* GGT was compared to that of the lid loop-deficient *B. subtilis* GGT and the lid loop-carrier *E. coli* GGT (Calvio et al., 2018) [1]. Here we report the experimental procedures for the synthesis of model substrates γ-glutamylglutamines through the method of the *N*-phtaloyl-L-glutamic acid anhydride and the spectral data of the synthetized compounds. The data obtained in the normalization procedure of the activities of the three enzymes are also reported.

**Specifications table**

**Table:** 

Subject area	*Chemistry*
More specific subject area	*Synthesis of model compounds as substrates for GGTs;Normalization of activities of different enzymes*
Type of data	*Reaction scheme, experimental procedures for synthesis, spectroscopic data*
How data was acquired	*Spectrophotometric reaction course monitoring (Jasco V-360 spectrophotometer interfaced with a PC running Spectra Manager software), NMR (Avance Bruker 400 instrument interfaced with a workstation running Topspin software package) and mass spectra (ThermoFinnigan LCQ Advantage mass spectrometer) for the characterization of the synthetized compounds.*
Data format	*Raw, filtered, analyzed*
Experimental factors	*N.A. (not applicable)*
Experimental features	*Oligo-γ-glutamyl derivatives were synthetized employing the method of N-phtaloyl-L-glutamic acid anhydride and purified through ion exchange column chromatography. Each compound was verified through*^*1*^*H and*^*13*^*C NMR and mass spectrometry.*
Data source location	*Department of Chemistry, University of Milano, via Golgi, 19 – 20133 Milano – Italy;Department of Biology and Biotechnology, University of Pavia, via Ferrata, 9 – 27100 Pavia - Italy*
Data accessibility	*This article*

**Value of the data**

• The synthesis of oligo-γ-glutamylglutamines is described.
• Spectroscopic data of the synthetized compounds are reported.
• The procedure for normalization of activities of different GGT enzymes is described.

## 1 Data

**Scheme 1:**
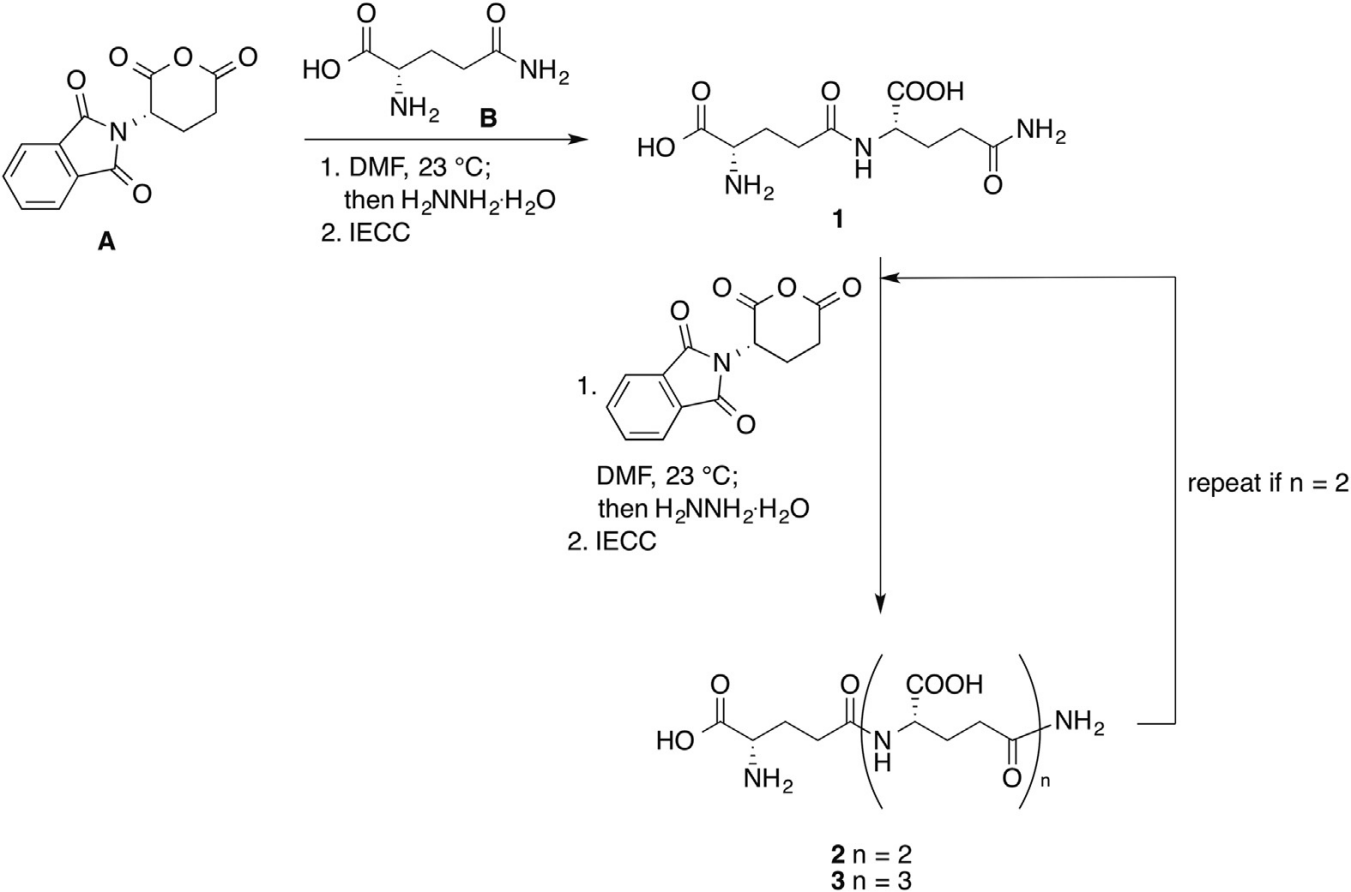
Synthesis of oligo-γ-glutamylglutamines **1**–**3**. *N*-phtaloyl-L-glutamic acid anhydride (A) and L-glutamine (B) were allowed to react in DMF at room temperature. At the end of the reaction, in situ removal of the *N*-phtaloyl protecting group was achieved by treatment with excess hydrazine hydrate. Hydrazinium salt of the product γ-glutamylglutamine **1** was precipitated together with phtalylhydrazide byproduct by adding ethanol at 0 °C and was collected by filtration. The hydrazinium salt of the product was taken up in water, leaving most of the insoluble phtalylhydrazide on the filter. The solution of the salt was basified to pH ca 9.5 and purified by ion exchange column chromatography (IECC) using Dowex 1 × 8 resin in the acetate form to give compound **1**. An aliquot of compound **1** was used as starting material in the obtainment of γ-glutamyl-γ-glutamylglutamine **2** following the same reaction scheme. An aliquot of compound **2** was used in turn for the synthesis of γ-glutamyl-γ-glutamyl-γ-glutamylglutamine **3**. (**A**) *N*-phtaloyl-L-glutamic acid anhydride; (**B**) L-glutamine; DMF = *N*,*N*-dimethylformamide; IECC = ion exchange column chromatography. **1** = γ-glutamylglutamine. **2** = γ-glutamyl-γ-glutamylglutamine. **3** = γ-glutamyl-γ-glutamyl-γ-glutamylglutamine.

The general procedure for the preparation of the model compounds:

• γ-glutamylglutamine (**1**),
• γ-glutamyl-γ-glutamylglutamine (**2**)
• γ-glutamyl-γ-glutamyl-γ-glutamylglutamine (**3**)

with the synthetic plan (Scheme 1) and the spectroscopic data of the compounds **1**–**3** are reported.

**Scheme 2:**
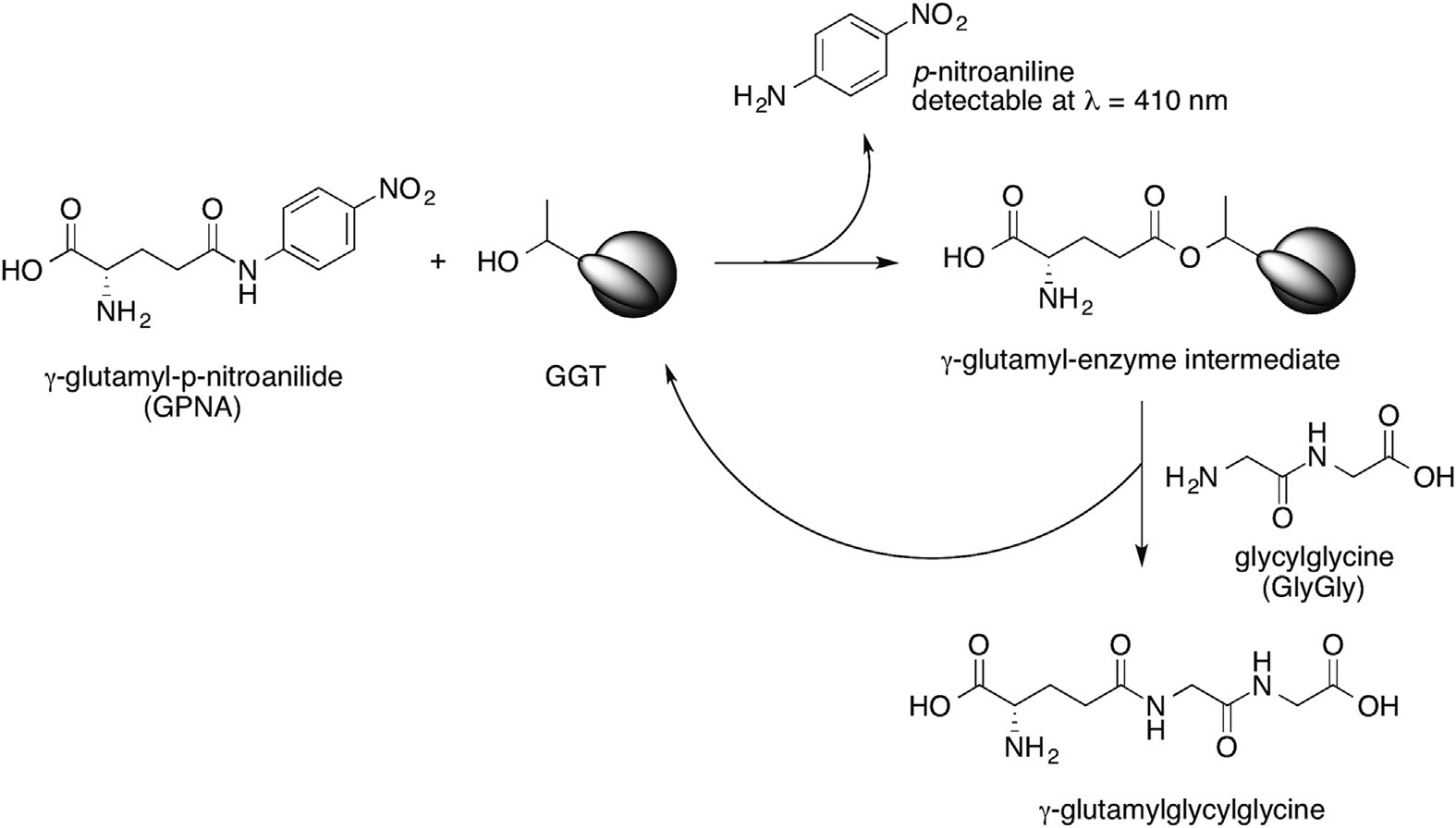
GGT-catalyzed reaction used in standard GGT activity test. Chromogenic γ-glutamyl-*p*-nitroanilide (GPNA) reacts as the donor substrate affording the γ-glutamyl-enzyme intermediate through reaction with the catalytically active threonine residue at the *N*-terminus of the small subunit of the enzyme. In this step *p*-nitroaniline (PNA) is liberated, which can be spectrophotometrically detected at 410 nm. The γ-glutamyl-enzyme intermediate is then resolved by nucleophilic attack of the free amino group of glycylglycine, present in solution in excess amount. The transpeptidation product γ-glutamylglycylglycine is formed and the enzyme is restored in its free state, able to start a new catalytic cycle. The reaction of the γ-glutamyl enzyme intermediate with a nucleophile is the rate-determining step of the process, thus the rate of liberation of PNA is usually considered a measure of the rate of the transpeptidase activity.

**Fig. 1:**
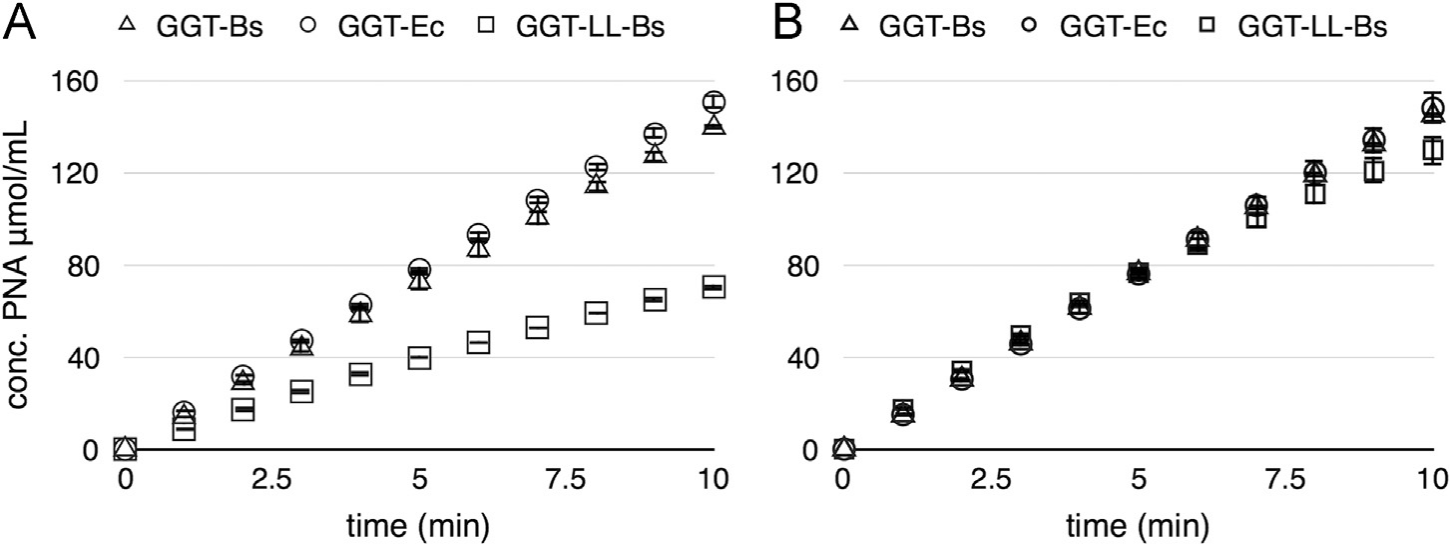
Enzyme activities measured for non-normalized and normalized enzymes. Time course of enzyme-catalyzed *p*-nitroaniline (PNA) liberation through transpeptidation reaction using GPNA as the donor and glycylglycine as the acceptor. (A) PNA liberation through enzyme-catalyzed reactions prior to enzyme standardization. (B) PNA liberation in reactions carried out with normalized enzymes.

The standard test used for measure the activity of GGTs is summarized in Scheme 2. Normalization of enzyme׳s activities, required to obtain comparable data when evaluating different GGT enzymes towards the same substrate [1], is reported in Fig. 1.

## 2 Experimental design, materials and methods

### 2.1 Synthesis of the oligomers of γ-glutamylglutamines **1**–**3**

#### 2.1.1 General

All reagents were from Sigma Aldrich and were used as received. DMF was distilled prior to use by standard methods. *N*-phtaloyl-L-glutamic acid anhydride was synthesized starting from *N*-phtaloyl-L-glutamic acid as previously reported [2].

TLC was carried out on silica gel F_254_ aluminum sheets using nBuOH-AcOH-water 4: 1: 1 as the eluent. TLC plates were inspected under an UV lamp (254 nm) and stained with 0.5% ninhydrin solution in ethanol and heating at 150 °C ca.

Dowex 1 × 8 ion exchange resin 200–400 mesh (Aldrich) in the acetate form was used for ion exchange column chromatography.

^1^H and ^13^C NMR spectra were acquired at 400.13 and 100.61 MHz, respectively, using a Bruker Avance 400 spectrometer interfaced with a workstation running Windows Operating System equipped with TopSpin Software package. Chemical shifts are given in ppm (δ) and are referenced to the signals of 3-(trimethylsilyl)propionic acid-2,2,3,3-d4 as external standard. Signal multiplicities in the ^13^C spectra were assigned on the basis of APT (attached proton test) experiments. Spectra analyses were carried out using inmr (www.inmr.net).

Mass spectra were acquired with a ThermoFinnigan LCQ Advantage mass spectrometer.

#### 2.1.2 General procedure for the synthesis of the oligomers of γ-glutamylglutamines **1**–**3**

1.05 eq of the compound to be γ-glutamylated was suspended in dry DMF (2 mL/mmol of starting material) in a two-necked round-bottom flask equipped with magnetic stirrer under a stream of nitrogen. *N*-phtaloyl-L-glutamic acid anhydride was added in one portion. The mixture was stirred at 21 °C until disappearance of the starting material (24–72 h), monitoring the progress of the reaction by TLC.

The reaction mixture was diluted with water (10% of the amount of DMF) and 3–6 eq. hydrazine hydrate, depending on the number of carboxyl groups in the product molecule, was added under vigorous stirring. Within 3 h a white, sticky solid precipitated. Ethanol was then added (2 mL/mmol of expected product) and stirring was continued at 0 °C for further 30 min in order to favor complete precipitation. The solid was collected by filtration on a sintered glass septum and washed in succession with cold ethanol, ethyl acetate and diethyl ether. The solid was then dried in a desiccator under reduced pressure without removing it from the septum.

The hydrazinium salt of the product was dissolved with small portions of water, leaving on the septum most of the phtalylhydrazide. The pH of the aqueous solution was adjusted to ca 9.5 with 1 M NaOH and charged onto a column of Dowex 1 × 8 ion exchange resin in the acetate form. The column was eluted with water until disappearance of hydrazine in the eluate (tested with 2% 4-dimethylaminobenzaldehyde in 10% HCl solution) and then with a scalar gradient of acetic acid (0.5, 1.0, 2.0, 3.0 M, three column volumes each). Eluate was collected in fractions; fractions containing the product were combined on the basis of TLC analysis and freeze-dried.

#### 2.1.3 γ-glutamylglutamine **1**

Obtained from glutamine and *N*-phtaloyl-L-glutamic acid anhydride in 23% yield after 72 h reaction time as a white, amorphous solid.

^1^H-NMR (400 MHz, D_2_O): δ 4.25 (dd, 1H, *J* = 9.0, 5.0 Hz, H-α Gln); 3.75 (t, 1H, *J* = 6.2 Hz, H-α Glu); 2.43 (t, 2H, *J* = 7.3 Hz, H-γ Glu); 2.15-2.05 (m, 3H, H-β Glu and H-β1 Gln); 1.92 (ddt, 1H, *J* = 14.3, 9.0 and 7.3 Hz, H-β2 Gln).

^13^C-NMR (100.1 MHz, D_2_O): δ 183.36, 179.38, 179.16, 176.35 (2×COOH; CONH; CONH_2_); 56.37 (C-α Glu); 55.38 (C-α Gln); 33.13 (C-γ Glu); 32.46 (C-γ Gln); 31.74 (C-β Glu); 28.47 (C-β Gln).

ESI-MS (negative mode): *m*/*z* 274.6 [M-H]^−^

#### 2.1.4 γ-glutamyl-γ-glutamylglutamine **2**

Obtained from γ-glutamylglutamine 1 and *N*-phtaloyl-L-glutamic acid anhydride in 75% yield after 24 h reaction time as a white, amorphous solid.

^1^H-NMR (400 MHz, D_2_O): δ 4.18 (t, 1H, J = 9.9 Hz, Hα-Glu); 4.17 (t, 1H, J = 9.0 Hz, Hα-Glu); 3.28 (t, 1H, J = 8.8 Hz, Hα-Gln); 2.41-2.31 (m, 6H); 2.21-2.12 (m, 2H); 2.00-1.79 (m, 4H).

^13^C-NMR (100 MHz, D_2_O): δ 178.60, 178.58, 178.40, 175.30, 175.25, 175.13 (COOH, CONH, CONH_2_); 55.02, 54.86, 54.73 (Cα); 32.47, 32.30, 31.80, 29.41, 27.82 (2×) (Cβ and Cγ).

ESI-MS (positive mode): *m/z* 427 [M+Na]^+^; 449 [M+2Na-H]^+^; 471 [M+3Na-2H]^+^, 493 [M+4Na-3H]^+^

#### 2.1.5 γ-glutamyl-γ-glutamyl-γ-glutamylglutamine **3**

Obtained from γ-glutamyl-γ-glutamylglutamine 2 and *N*-phtaloyl-L-glutamic acid anhydride in 53% yield after 48 h reaction time as a white, amorphous solid.

^1^H-NMR (400 MHz, D_2_O): δ 4.33-4.27 (m, 3H, Hα-Glu); 3.84-3.82 (m, 1H, Hα-Gln); 2.49-2.45 (m, 2H); 2.40-2.30 (m, 6H); 2.21-2.09 (m, 5H); 1.99-1.88 (m, 3H).

^13^C NMR (100 MHz, D_2_O): δ 178.61, 178.37, 178.30; 175.12 (2×), 174.37 (2×) (COOH, CONH, CONH_2_); 54.83, 54.66, 54.58, 54.41 (Cα); 32.40, 32.33, 31.75, 31.73, 27.71, 27.60, 26.39 (2×) (Cβ and Cγ)

ESI-MS (negative mode): *m*/*z* 532.5 [M-H]^−^

### 2.2 Enzymes standardization

Enzyme activity assays were carried out using γ-glutamyl-*p*-nitroanilide 0.25 mM and glycylglycine 2 mM in 0.1 M NaHCO_3_ solution, pH 8.2. Reactions were initiated by adding 20 μL of an arbitrarily diluted enzyme solution. Final volume was 2 mL. Absorbance was measured at *λ* = 410 nm for 10 min, recording data every min. On the basis of the measured activities (Fig. 1A), stock enzymes’ solutions were differentially diluted in order to have the same amount of enzyme units and activities were again verified through the same assay (Fig. 1B).

## Figures and Tables

**Fig. S1:**